# Vitamin D deficiency as a risk factor for dementia: a systematic review and meta-analysis

**DOI:** 10.1186/s12877-016-0405-0

**Published:** 2017-01-13

**Authors:** Isolde Sommer, Ursula Griebler, Christina Kien, Stefanie Auer, Irma Klerings, Renate Hammer, Peter Holzer, Gerald Gartlehner

**Affiliations:** 1 Department for Evidence-based Medicine and Clinical Epidemiology, Danube University Krems, Dr.-Karl-Dorrek-Straße 30, 3500 Krems, Austria; 2 Cochrane Austria, Danube University Krems, Krems, Austria; 3 Department for Clinical Neurosciences and Preventive Medicine, Danube University, Krems, Austria; 4 MAS Alzheimerhilfe, Bad Ischl, Austria; 5 Institute of Building Research & Innovation ZT-GmbH, Vienna, Austria; 6 RTI-UNC Evidence-based Practice Center, Research Triangle Institute International, North Carolina, USA

**Keywords:** Dementia, Vitamin D, Systematic review, Meta-analysis

## Abstract

**Background:**

Sunlight exposure and high vitamin D status have been hypothesised to reduce the risk of developing dementia. The objective of our research was to determine whether lack of sunlight and hypovitaminosis D over time are associated with dementia.

**Methods:**

We systematically searched MEDLINE (via PubMed), Cochrane Library, EMBASE, SCOPUS, Web of Science, ICONDA, and reference lists of pertinent review articles from 1990 to October 2015. We conducted random effects meta-analyses of published and unpublished data to evaluate the influence of sunlight exposure or vitamin D as a surrogate marker on dementia risk.

**Results:**

We could not identify a single study investigating the association between sunlight exposure and dementia risk. Six cohort studies provided data on the effect of serum vitamin D concentration on dementia risk. A meta-analysis of five studies showed a higher risk for persons with serious vitamin D deficiency (<25 nmol/L or 7–28 nmol/L) compared to persons with sufficient vitamin D supply (≥50 nmol/L or 54–159 nmol/L) (point estimate 1.54; 95% CI 1.19–1.99, I^2^ = 20%). The strength of evidence that serious vitamin D deficiency increases the risk of developing dementia, however, is very low due to the observational nature of included studies and their lack of adjustment for residual or important confounders (e.g. ApoE ε4 genotype), as well as the indirect relationship between Vitamin D concentrations as a surrogate for sunlight exposure and dementia risk.

**Conclusions:**

The results of this systematic review show that low vitamin D levels might contribute to the development of dementia. Further research examining the direct and indirect relationship between sunlight exposure and dementia risk is needed. Such research should involve large-scale cohort studies with homogeneous and repeated assessment of vitamin D concentrations or sunlight exposure and dementia outcomes.

**Electronic supplementary material:**

The online version of this article (doi:10.1186/s12877-016-0405-0) contains supplementary material, which is available to authorized users.

## Background

With life expectancy on the rise throughout the world and almost 900 million people 60 years old or over [1], the prevalence of neurodegenerative diseases such as dementia is increasing. Dementia is characterized by multiple cognitive deficits that include impairment in memory [2] and encompasses four subtypes (Alzheimer’s disease, vascular dementia, Lewy body dementia, and frontotemporal dementia), which are each associated with specific neurological features [3]. In 2015, 46.8 million people worldwide were living with dementia. This number is predicted to double every 20 years, until at least 2050 [1], even though evidence from European countries shows stable age-specific prevalence and incidence rates over time [4]. An increase in dementia seems unavoidable due to the fact that people live longer. Based on data from the Framingham study, the lifetime risk of dementia at age 65 is 22% for women and 14% for men [5]. Since the causes of dementia remain unknown and no cure for the disease has been found [6], researchers are intensely searching for preventive interventions to delay the onset of dementia. Several risk factors have been identified but the evidence for these is variable. Biological factors possibly associated with dementia risk include old age, female sex, and Apolipoprotein E (ApoE) ε4 genotype. Social factors that may contribute to risk include low education and low socioeconomic status. Lifestyle characteristics such as alcohol abuse, smoking, and reduced physical activity could also play a role. Medical risk factors such as high blood pressure, high cholesterol, overweight, diabetes, and cardiovascular diseases are also believed to be contributing factors [7].

In recent years, evidence has linked a lack of vitamin D not only to its known effects on calcium and bone metabolism, but also to neurocognitive decline [8]. About 90% of vitamin D is produced in the epidermis from 7-dehydrocholesterol (7-DHC) as a reaction to sunlight (solar ultraviolet B radiation; 290–315 nm) [9]. Factors that limit the cutaneous production of vitamin D3 include higher latitude, covering of skin, lack of outdoor activities, sunscreen use, old age, female sex, and darker skin pigmentation [10]. In an assessment derived from published studies, Holick [9] has estimated that due mainly to lack of sunlight exposure, approximately one billion people worldwide have inadequate vitamin D levels (as defined by a 25-hydroxyvitamin D or 25(OH)D, the primary circulating form of vitamin D in the serum, level of <75 nmol/L). In addition to sunlight, another important source of vitamin D is nutrition. Persons residing in regions where sunlight is reduced like in northern Europe need to include foods rich in vitamin D such as fatty fish or vitamin D fortified foods in their diets [10].

Recent systematic reviews and meta-analyses from cross-sectional analyses suggest that low serum vitamin D concentrations may be associated with Alzheimer’s disease and other forms of dementia and cognitive impairment [11, 12]. However, other systematic reviews (for example, Barnard and Colon-Emeric [13]) could not find an association between cognitive function (measured with the Mini-Mental State Examination [MMSE]) and 25(OH)D concentration. Results of systematic reviews, however, can vary due to differences in search strategies, inclusion criteria, statistical analysis techniques, and adjustment of confounding factors. Within the spectrum of observational studies, longitudinal studies may be more valid than other observational study designs because they commonly take confounding factors into account and also give insight into the temporal order of cause and effect. The objective of our study, therefore, was to focus on longitudinal studies to systematically and objectively evaluate the influence of sunlight exposure or vitamin D on dementia risk.

## Methods

This systematic review was prospectively registered in PROSPERO (International Prospective Register of Systematic Reviews) [14] [CRD42014010199]. Throughout this manuscript, we followed the PRISMA (Preferred Reporting Items for Systematic Reviews and Meta-Analyses) statement [15] to report this systematic review.

### Literature search

We searched MEDLINE (via PubMed), Cochrane Library, EMBASE, SCOPUS, Web of Science, and ICONDA from January 1990 to October 2015 to identify relevant publications. We further searched for grey literature using the PsycInfo database for dissertations and theses, the SCOPUS database for conference proceedings, and the Open Grey database. We limited searches to human populations and English or German language. An experienced information specialist developed an appropriate search strategy using a combination of Mesh (Medical subject headings) terms and free-text key words (dementia, sunlight or vitamin D) and ran the searches. The detailed search strategy is presented online in the Additional file 1. Additionally, we complemented electronic searches by checking reference lists from pertinent studies and reviews and contacting experts for their suggestions of relevant articles. We imported all citations into a reference managing database (Endnote X · 6 · 0 · 1) and deleted duplicate publications.

### Inclusion criteria

We included randomised and non-randomised controlled trials, prospective cohort studies, nested case-control studies and systematic reviews on longitudinal studies that investigated the effect of sunlight exposure or vitamin D serum concentrations (as surrogate parameter for sunlight) on prevalence or incidence of dementia including Alzheimer’s disease, vascular dementia, frontotemporal dementia, and Lewy body dementia (diagnosis based on validated measurement scales) among adults. We did not consider any studies on mild cognitive impairments or any intervention studies on vitamin D supplementation and dementia risk for inclusion. The scientific expert panel for this review deemed prevalence or incidence of dementia as critical outcomes for decision-making.

### Study selection

Two reviewers independently screened abstracts and full-texts against pre-specified criteria. They resolved discrepancies about inclusion or exclusion by consensus or by involving a third reviewer. Studies that were only published as abstract were excluded.

### Data extraction

We designed, pilot-tested, and used standardised data extraction forms to gather pertinent information from each study. Two trained reviewers extracted data relating to: a) study information (author, publication year, funding, location/setting); b) observation period; c) study design; d) sample size; e) outcome measurement; f) description of study population including individual characteristics such as age, gender, and type of dementia; and g) results of the study. If articles did not provide enough information to extract relevant data, authors were contacted in an attempt to acquire additional information. A second reviewer checked all abstracted data for completeness and accuracy.

### Study quality

We evaluated the methodological quality (risk of bias) of studies using a modified version of the Newcastle-Ottawa Scale (NOS) for observational studies [16]. Two independent reviewers assessed the risk of bias for each study. Disagreements between the two reviewers were resolved by discussion and consensus or by consulting a third member of the team. The result was an overall risk-of-bias rating of each study classed as low, unclear, or high risk of bias.

### Data synthesis

We performed a random effect meta-analysis using the generic inverse variance model to synthesise effect estimates of studies that were similar with respect to exposure classification. In case of incongruous exposure categories, we contacted study authors asking to reanalyse the data using ≥50 nmol/L [no deficiency or sufficient supply], ≥25 to <50 nmol/L [insufficiency], and <25 nmol/L [serious deficiency] serum vitamin D concentrations for classification. In absence of an agreed definition, these cut-offs are commonly used by experts and reflect vitamin D recommendations by several organisations. They were set with regard to prevention of rickets and/or symptomatic osteomalacia (<25 nmol/L) and guarantee of sufficient supply of vitamin D for almost the whole population (97.5%) (≥50 nmol/L) [8]. A separate analysis of the data using fixed effect meta-analysis yielded similar results. We tested for heterogeneity with Cochrane’s Q test and quantified its magnitude using I^2^. The small number of studies identified precluded a reliable visual assessment of publication bias. We conducted all statistical analyses using Comprehensive Meta-Analysis version 3. The results of studies not suitable for inclusion in the meta-analysis are reported narratively.

### Ratings of quality of evidence

We graded the quality of the available evidence in a four-part hierarchy according to the GRADE scheme (Grading of Recommendations Assessment, Development and Evaluation) [17]. GRADE assesses the quality of evidence using four grades: high, moderate, low, and very low [18]. Observational studies always start with a rating of low quality of evidence because of the risk of residual confounding but can be upgraded for large treatment effects, dose effect gradients, or if apparent confounding would reduce the observed effect [19]. Criteria for downgrading the quality of evidence are risk of bias [20], imprecision [21], inconsistency [22], indirectness [23], and publication bias [24]. We dually evaluated the overall quality of evidence for each outcome viewed as “critical” for decision-making by the scientific expert panel. We reconciled all disagreements in grades through consensus discussion.

## Results

### Study characteristics

**Fig. 1:**
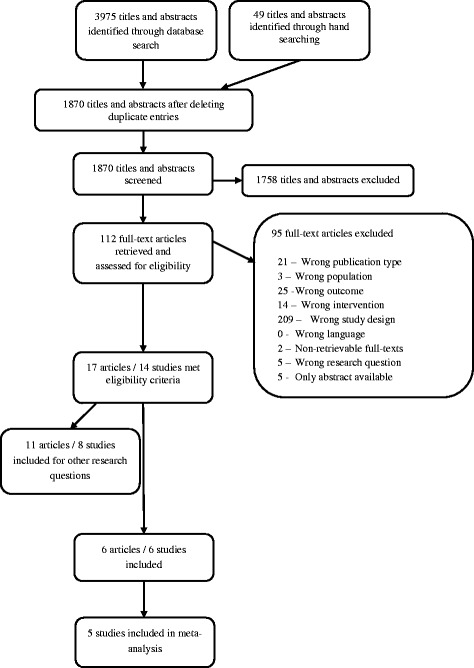
Flow diagram for the study selection process

We identified a total of 1870 citations from searches and reviews of reference lists after removal of duplicates and assessed 112 full-text reviews for eligibility as part of a larger research report. Overall, 17 articles met the inclusion criteria for the larger research report, of which we included six for our research question (see Fig. 1).

Because we could not identify any direct evidence on exposure to sunlight and risk of developing dementia, we focused our review on indirect evidence from studies using vitamin D status as a surrogate parameter. The six studies [25–30] that fulfilled the criteria of indirect evidence were cohort studies providing data on 18,974 adults. There was one study that we rated as high risk of bias [25], all others received an unclear risk-of-bias rating [26–30]. Reasons for the high risk of bias-rating were the unjustified selection of study participants from one out of five possible settings, inadequate assessment of possible confounders, as well as a dropout rate of 56%.

The characteristics of all included studies [25–30] are displayed in Table 1. Studies showed differences in terms of study population, sample size, participants’ age, follow-up, vitamin D exposure classification, methods for serum vitamin D measurement, adjustment of potential confounding factors, and timing and criteria used to diagnose dementia. Diagnosis of dementia was made according to the International Statistical Classification of Diseases and Related Health Problems (ICD 8, 9 or 10) [26–30], the Diagnostic and Statistical Manual of Mental Disorders (DSM-IV or IV-TR), in combination with other criteria [26–30] or the criteria of the National Institute of Neurological and Communicative Disorders and Stroke and the Alzheimer’s Disease and Related Disorders Association alone [29]. It was either performed by experts [26–30] or taken from hospital discharge records and patient or death registries [26–30]. All studies were prospective cohort studies, apart from Knekt et al.’s [28], which was a retrospective investigation. Half of the studies received public research funding [25, 28, 30] and half of them a mix of public and private research funding [26, 27, 29]. They were conducted in the United States [29, 30], Finland [28], Denmark [26], Switzerland [27], and France [25].

**Table 1:** Characteristics of included studies

Reference, study design, study name (period)	Location, setting, funding	Population, baseline characteristics	Exposure (measurement), collection period	Follow-up period	Risk of bias
Afzal et al., 2014 [1], prospective cohort study, Copenhagen City Heart Study (1981–1983)	Denmark, general population (population register), public research funding and material sponsorship from Diarosin Liasion	10,186 individuals without dementia Women % (n): 56.1% (5718) Age in y (median, range): total: n.s.; E1: 57 (47–64), E2: 58 (49–65), E3: 58 (50–65)	Plasma 25(OH)D measurement: DiaSorin Liaison 25(OH)D total assay (Immunoassay) Sample collection: 1981–1983 stored until 2009 to 2010 E1: no vit D deficiency: ≥50 nmol/L (>20 ng/mL)^a^ E2: vit D deficiency: ≥25 to <50 nmol/L (≥10 to 20 ng/mL) E3: serious vit D deficiency: <25 nmol/L (<10 ng/mL)	Median 21 y (Range: 0.03–30 y) until diagnosis of AD, vascular dementia, death, emigration or May 2011	unclear
Annweiler et al., 2011 [2], prospective cohort study, EPIDémiologie de l’OStéoporose (EPIDOS) study Toulouse (1992–1994)	France, general population, public research funding	40 women without dementia from the EPIDOS Toulouse study Women (n): 100% (40) Age in y (median, 25./75. percentile): 78.4 (76.4/82.0)	Serum 25(OH)D measurement: Radioimmunoassay Sample collection: 1992–1994 E1: ≥25 nmol/L (≥10 ng/mL) E2: <25 nmol/L (<10 ng/mL)	7 y	high
Graf et al. 2014 [3], prospective cohort study	Switzerland, geriatric hospital, public research funding and material sponsorship from AstraZeneca Switzerland (2004–2005)	246 patients, of these 200 cognitively normal, 46 with mild cognitive impairment (MCI) Women % (n): 75.6% (147 cognitively normal and 39 MCI) Age in y (mean, SD): total: n.s.; cognitively normal: 84.4 (7.1), MCI: 85.3 (6.6)	Plasma 25(OH)D measurement: Electrochemiluminescence-Immunoassay Sample collection: 2004–2005 E1: optimal vit D status: ≥75 nmol/L (≥30 ng/mL)^a^ E2: sub-optimal vit D status: 50–75 nmol/L (20–30 ng/mL) E3: vit D insufficiency: 25–49,9 nmol/L (10–19,96 ng/mL) E4: vit D deficiency: <25 nmol/L (<10 ng/mL) Reclassification for Meta-Analysis: e1: no vit D deficiency: ≥50 nmol/L (≥20 ng/mL) e2: vit D deficiency: ≥25 to <50 nmol/L (≥10 to 20 ng/mL) e3: serious vit D deficiency: <25 nmol/L (<10 ng/mL)	2 y	unclear
Knekt et al., 2014 [4], retrospective cohort study, Mini Finland Health Survey (1978–1980)	Finland, general population (population register), public research funding	5010 subjects without hospitalisation due to dementia Women % (n): 54.7% (2738) Age in y (median): total: n.s.; E1: 54, E2: 55, E3: 56, E4: 59	Serum 25(OH)D measurement: Radioimmunoassay (DiaSorin) Sample collection: 1978–1980 stored until 2003 E1: 4. quartile: 54–159 nmol/L (21.6–63.6 ng/mL)^a^ E2: 3. quartile: 40–53 nmol/L (16–21.2 ng/mL) E3: 2. quartile: 29–39 nmol/L (11.6–15.6 ng/mL) E4: 1. quartile: 7–28 nmol/L (2.8–11.2 ng/mL)	17 y	unclear
Littlejohns et al., 2014 [5], prospective cohort study, Cardiovascular Health Study (1992–1993)	USA, 4 communities, ambulatory participants, private and public research funding,	1658 subjects without dementia, cardiovascular diseases or stroke Women % (n): 69.2% (1148) Age in y (median, SD): 73.6 (4.5)	Serum 25(OH)D measurement: LC-MS/MS Sample collection: 1992–1993 stored until 2008 E1: no vit D deficiency: ≥50 nmol/L (≥20 ng/mL) E2: vit D deficiency: ≥25 to <50 nmol/L (≥10 to 20 ng/mL) E3: serious vit D deficiency: <25 nmol/L (<10 ng/mL)	Average 5.6 y (SD 1.6)	unclear
Schneider et al. 2014 [6], prospective cohort study, Atherosclerosis Risk in Communities (ARIC) Brain MRI Study (1993–1995)	USA, general population from 2 regions, public research funding	1652 subjects (white or black ethnic background) without hospitalisation due to dementia, cardiovascular diseases or stroke Women % (n): 60.3% (996) Age in y (mean, SD): total: 62 (n.s.).; E1: Whites 63.1 (4.3); Blacks 62.2 (4.4), E2: Whites 63.3 (4.5); Blacks 61.4 (4.6), E3: Whites 62.9 (4.4); Blacks 61.0 (4.5)	Plasma 25(OH)D measurement: LC-MS/MS Sample collection: 1993–1995 stored until 2012 E1: highest tertile Whites ≥70.8 nmol/L^a^ (≥28.3 ng/mL); Blacks ≥48.3 nmol/L (≥19.3 ng/mL); E2: middle tertile: Whites 54.5 to <70.8 nmol/L (21.8 to <28.3 ng/mL); Blacks 35.0 to <48.3 nmol/L (14.0 to <19.3 ng/mL) E3: lowest tertile: Whites <54.5 nmol/L (<21.8 ng/mL); Blacks <35.0 nmol/L (<14.0 ng/mL) Reclassification for Meta-Analysis: e1: no vit D deficiency: ≥50 nmol/L (≥20 ng/mL) e2: vit D deficiency: ≥25 to <50 nmol/L (≥10 to 20 ng/mL) e3: serious vit D deficiency: <25 nmol/L (<10 ng/mL)	Median 16.6 y	unclear

*Abbreviations*: *AD* Alzheimer’s disease, *nmol/L* nanomoles per litre, *ng/mL* nanograms per millilitre, *SD* standard deviation, *Vit D* Vitamin D, *n* number, *n.s.* not specified, *E or e* exposure, *y* years, *LC-MS* liquid chromatography-tandem mass spectrometry, *MCI* mild cognitive impairment

^a^To convert 25(OH)D to nanomoles per litre from nanograms per litre, multiply values by 2.5

### Incidence of dementia

We conducted a meta-analysis (random effects model) to derive a pooled estimate for dementia risk. Despite incongruent use of vitamin D exposure categories across publications but with the help of some study authors [27, 30] who provided unpublished data, we were able to combine data from a total of 18,933 persons included in five studies [26–30] (Table 2). We did not include the study of Annweiler et al. [25] in the meta-analysis as it used different vitamin D cut-offs to make comparisons between subjects (<25 nmol/L vs ≥25 nmol/L).

**Table 2:** Results of included studies

Study, year, study design	Population	Outcome measures (assessment methods)	Confounder (measured)	Results	Risk of bias
Afzal et al., 2014 [1], prospective cohort study	E1: 3715 E2: 4087 E3: 2384	Incidence of AD or dementia (ICD 8th and 10th edition diagnoses entered in the national Danish Patient Registry and the national Danish Causes of Death Registry)	Gender, age, smoking status, BMI, leisure time and work-related physical activity, income level, education, diabetes mellitus, hypertension, alcohol consumption, cholesterol, creatinine, month of blood sample, seasonal adjusted vit D concentrations	418 subjects developed AD and 92 subjects vascular dementia, 14 subjects had both diagnoses. Risk of developing dementia: Analysis adjusted for all measured confounders: AD: E1 = reference, E2: HR = 1.23 (95% CI 0.97–1.55), E3: HR = 1.29 (95% CI 1.01–1.66) (p = 0.03) Vascular dementia: E1 = reference, E2(<50 nmol/L or 20 ng/mL): HR = 1.22 (95% CI 0.79–1.87), (p = 0.42) Combined: E1 = reference, E2: HR = 1.24 (95% CI 1.00–1.54), E3: HR = 1.27 (95% CI 1.01–1.60) (p = 0.02)	unclear
Annweiler et al., 2011 [2], prospective cohort study	E1: 33 (subtle cognitive impairment [2]) E2: 7 (subtle cognitive impairment [1])	Incidence of dementia (diagnosed by experts, according to DSM IV, NINCDS-ADRDA)	Subtle cognitive impairment at baseline, presence of cardiovascular risk factors at baseline (age >85 years, hypertension, diabetes mellitus, BMI >25, lack of physical activity, smoking), diagnosis of Parkinson’s disease at baseline	10 women developed dementia, 4 of these AD E1: 3 E2: 7 (4 AD) Risk of developing Non-Alzheimer’s dementia Analysis adjusted for all measured confounders: E1 = reference E2: OR = 19.57 (95%CI 1.11–343.69) Risk of developing AD Unadjusted analysis: E1 = reference E2: OR = 1.06 (95%CI 0.97–1.15).	high
Graf et al. 2014 [3], prospective cohort study	E1: 15 (cognitively normal [11], MCI [4]) E2: 33 (cognitively normal [27], MCI [6] E2: 58 (cognitively normal [52]; MCI [6]) E3: 140 (cognitively normal [110, MCI [30])	Incidence of dementia (diagnosed by experts, validated cognitive scales, DSM IV-TR, NINCDS-ADRDA, ADDTC, and NINDS-AIREN)	Gender, age, education level, basic (BADL) and instrumental (IADL) activities of daily living, comorbidities (CIRS), calcaemia, Vit B12 status, ApoE Eε4 genotype, mini nutritional assessment, albuminaemia, BMI	46 subjects developed dementia, 28 cognitively normal subjects and 18 with MCI. Analysis adjusted for all measured confounders: E1 = reference E2: RR = 2.87 (95% CI 0,36–22,77) E3: RR = 6.18 (95% CI 0,87–43,76) E4: RR = 2.85 (95% CI 0,45–17,95) Reclassification for Meta-Analysis: Analysis adjusted for all measured confounders: e1 = reference e2: RR = 4.55 (95% CI 1.04–19.82) e3: RR = 1.35 (95% CI 0.39–4.61)	unclear
Knekt et al., 2014 [4], retrospective cohort study	E1: 1240 E2: 1258 E3: 1216 E4: 1296	Incidence of dementia leading to hospitalisation (ICD 8 from the nationwide Finnish hospital discharge register or death certificates from Statistics Finland)	Gender, age, month of blood sample, education level, marital status, leisure time physical activity, smoking status, BMI, alcohol consumption, hypertension, plasma fasting glucose concentration, serum triglyceride concentration, serum total cholesterol concentration	151 subjects developed dementia, 34 men and 117 women. E1: 21 (m = 13, f = 8) E2: 33 (m = 12, f = 21) E3: 37 (m = 5, f = 32) E4: 60 (m = 13, f = 47) Analysis adjusted for all measured confounders: Men: E1: HR = 0.74 (95% CI 0.29–1.88) E2: HR = 0.63 (95% CI 0.25–1.56), E3: HR = 0.41 (95% CI 0.14–1.19), E4 = reference Women: E1: HR = 0.33 (95% CI 0.15–0.73) E2: HR = 0.60 (95% CI 0.34–1.06), E3: HR = 0.90 (95% CI 0.56–1.44), E4 = reference Combined: E1: HR = 0.48 (95% CI 0.28–0.84) E2: HR = 0.62 (95% CI 0.39-1.00), E3: HR = 0.75 (95% CI 0.49-1.14), E4 = reference	unclear
Littlejohns et al., 2014 [5], prospective cohort study	E1: 1169 E2: 419 E3: 70	Incidence of dementia (diagnosed by experts, annual cognitive assessments, NINCDS-ADRDA)	Age, season of vit D collection, education, gender, BMI, smoking, alcohol consumption, depressive symptoms, diabetes, hypertension, ethnicity, income, occupation	171 subjects developed dementia, 102 of these AD E1: n.s. E2: n.s. E3: n.s. Analysis adjusted for age, season of vit D collection, education, gender, BMI, smoking, alcohol consumption, depressive symptoms: Dementia: E1 = reference, E2: HR = 1.53 (95% CI 1.06–2.21), E3: HR = 2.25 (95% CI 1.23–4.13) (p = 0.002) AD: E1 = reference, E2: HR = 1.69 (95% CI 1.06–2.69), E3: HR = 2.22 (95% CI 1.02–4.83) (p = 0.008) Similar results for analysis that additionally adjusted for diabetes and hypertension (data not shown).	unclear
Schneider et al. 2014 [6], prospective cohort study	E1: Whites 285; Blacks 267 E2: Whites 283; Blacks 272 E3: Whites 284; Blacks 261	Incidence of AD or dementia leading to first hospitalisation (ICD 9 from hospital discharge records)	Gender, age, education, income, smoking, alcohol consumption, physical activity, BMI, waist circumference, use of vit D supplements, diabetes, hypertension, use of hypertension medication, cholesterol, estimated glomerular filtration rate, calcium status, phosphate, PTH, season adjusted vit D concentrations	145 subjects developed AD or dementia. E1: Whites 18; Blacks 23 E2: Whites 31; Blacks 24 E3: Whites 24; Blacks 25 Analysis adjusted for age, gender, education, income, physical activity, smoking, alcohol consumption, BMI, wait circumference, use of vit D supplements: Whites: E1 = reference, E2: HR = 1.74 (95% CI 0.95–3.18), E3: HR = 1.32 (95% CI 0.69–2.55) Blacks: E1 = reference, E2: HR = 1.22 (95% CI 0.68–2.19), E3: HR = 1.53 (95% CI 0.84–2.79) Reclassification for Meta-Analysis: 145 subjects developed AD or dementia. e1: 75 out of 876 e2: 63 out of 694 e3: 7 out of 82 Analysis adjusted for age, gender, education, income, physical activity, smoking, alcohol consumption, BMI, waist circumference, use of vit D supplements: e1 = reference e2: HR = 1.22 (95% CI 0.85-1.74) e3: HR = 1.44 (95% CI 0.65-3.21)	unclear

*Abbreviations*: *AD* Alzheimer’s diseases, *APOE ε4* apolipoproteine E *ε*4 genotype, *ADDTC* Alzheimer’s Disease Diagnostic and Treatment Centres, *BMI* Body-Mass Index, *BADL* Basic Activities of Daily Living, *CIRS* Cumulative Index Rating Scale, *DSM-IV-(TR)* Diagnostic and Statistical Manual of Mental Disorders, Fourth Edition, (Text Revision), *E or e* exposure, *f* female, *HR* hazard ratio, *IADL* Instrumental Activities of Daily Living, *ICD* International Classification of Diseases, *y* years, *n.s.* not specified, *CI* confidence interval, *m* male, *n* number, *NINCDS-ADRDA* National Institute of Neurological and Communicative Disorders and Stroke and the Alzheimer’s Disease and Related Disorders Association, *NINDS-AIREN* National Institute of Neurological Disorders and Stroke and Association Internationale pour la Recherché et l’Enseignement en Neurosciences, *ADDTC* Alzheimer’s Disease Diagnostic and Treatment Centers, *p* p-value, *PTH* parathormone, *RR* relative risk, *SD* standard deviation, *vit D* vitamin D, *vit B12* vitamin B12, *OR* odds ratio, *MCI* mild cognitive impairment

**Fig. 2:**
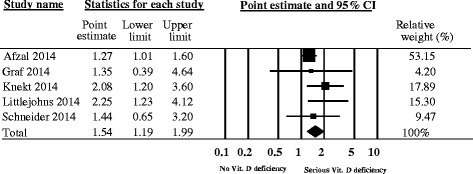
Meta-Analysis of the incidence of dementia in relation to vitamin D status. Random effects meta-analysis; I^2^ = 20%

The meta-analysis across all five studies [26–30] demonstrated a statistically significantly higher dementia risk in persons with serious vitamin D deficiency (<25 nmol/L or 7–28 nmol/L) than in persons with sufficient vitamin D supply (≥50 nmol/L or 54–159 nmol/L) (Point estimate = 1.54; 95% confidence interval [CI]: 1.19 to 1.99, see Fig. 2). Translated into absolute numbers, 28 (at least 10 but up to 50 more) out of 1000 people with serious vitamin D deficiency would develop dementia compared with 1000 people with sufficient D levels over 18.03 years. Heterogeneity of the meta-analysis was low (I^2^ = 20%) and was examined using sensitivity analyses. We examined factors such as use of different cut-off points to categorise vitamin D exposure, age, lack of adjustment for seasonal vitamin D change in the analysis, use of dementia incidence leading to hospitalisation as endpoint, and vitamin D assessment by liquid chromatography-tandem mass spectrometry on the robustness of the results of the meta-analysis. The analyses showed only minor variations and thus confirmed the robustness of the results (see Additional file 2: Figures S1–S5).

The study of Annweiler et al. [25] comprised only 40 participants. Findings, however, were consistent with those of our meta-analysis. Results showed a statistically significant association between vitamin D deficiency and non-Alzheimer’s dementia (adjusted OR = 19.57; 95% CI 1.11 to 343.69). The association between vitamin D and Alzheimer’s disease (unadjusted OR = 1.06; 95% CI 0.97 to 1.15; adjusted OR not reported) was not statistically significant.

The strength of evidence that vitamin D increases the risk of developing dementia is very low (Table 3). This is because the evidence exclusively relies on observational studies which did not equally consider all important confounders (e.g. ApoE ε4 genotype) and which assessed the indirect relationship between vitamin D as surrogate for sunlight exposure and dementia risk.

**Table 3:** Summary of findings for influence of sunlight exposure on risk of dementia

Population: General population aged between 54 and 85.3 years (median/mean) Settings: Health examinations, geriatric hospitals Exposure/Risk: Vitamin D deficiency Comparison: No vitamin D deficiency (reference group)						
Outcomes	Comparative risk (95% CI) and narrative results		Relative effect (95% CI)	Number of participants (number of studies)	Quality of evidence (GRADE)	Comments
	No exposure (no vitamin D deficiency)	Exposure (vitamin D deficiency)				
Incidence of dementia (Results meta-analysis) Follow-up:18.03 years (weighted mean)	Study population (≥50 nmol/L or ≥ 54–159 nmol/L)	(<25 nmol/L or 7–28 nmol/L)	Point (raw) 1.54 (1.19 to 1.99)	18 639 subjects (5 studies)	⊕⊝⊝⊝ Very low^a,b^	
Incidence of dementia (Narrative results) Follow-up: 7 years	Study population (≥25 nmol/L)	(<25 nmol/L)	OR 19.57 (1.11 to 343.69)	40 subjects (1 study)		

*Abbreviations*: *CI* confidence interval, *nmol/L* nanomoles per Litre, *OR* odds ratio

^a^Risk of bias: Adjustment for confounders varies across study groups, Apo E *ε*4 genoytpe only considered in one study

^b^Indirectness: no study investigated the direct relationship between solar radiation and dementia, vitamin D is a surrogate parameter

## Discussion

To our knowledge, this is the first systematic review that evaluated the influence of vitamin D serum concentrations on dementia risk using longitudinal studies. Despite a comprehensive search of the literature we were not able to identify any study that addressed the direct relationship between sunlight exposure and dementia risk. The meta-analysis of five out of six included studies with data from a total of 18,639 participants showed that persons with serious vitamin D deficiency have a statistically significant higher relative risk of 1.54 to develop dementia than persons with sufficient vitamin D supply. Our findings seem to suggest that vitamin D deficiency increases the risk of dementia. However, the quality of the evidence is very low because of the observational nature of included studies, the fact that not all studies considered important confounders equally, and the use of vitamin D as surrogate for sunlight exposure. As a consequence, a causal effect of vitamin D deficiency on dementia cannot be assumed with certainty.

Research has still not identified the physiological mechanisms underlying the potential effect of vitamin D deficiency on dementia risk but several candidate mechanisms have been identified. Evidence from animal studies [31] suggests that vitamin D deficiency may impair neurophysiological functioning causing anatomical and behavioural adverse effects. In a landmark publication, Eyles et al. [32] provide evidence that the 1,25-dihydroxyvitamin D3 receptor (VDR) is wide-spread in the human brain. Mapping their distribution in the brain area, they found the strongest immunohistochemical presence of both the receptor and the connected enzyme (1alpha-OHase) in the hypothalamus and in large neurons of the substantia nigra. Based on the observed distribution of the VDR and 1alpha-OHase, they concluded that vitamin D may act in a manner similar to neurosteroids [32]. The neurosteroid actions of vitamin D encompasses regulation of calcium homeostasis, β-amyloid deposition, antioxidant and anti-inflammatory properties, and potential protection against neurodegenerative processes associated with Alzheimer’s disease and cognition [33].

Two previous systematic reviews have investigated the association between vitamin D intake and dementia risk [11, 12], focussing on Alzheimer’s disease as the most common subtype of dementia [34]. Because at the time of their literature searches large cohort studies were not published yet, they based their conclusions on cross-sectional analyses from case-control studies mainly. Both systematic reviews found lower serum vitamin D concentration in cases with Alzheimer’s diseases compared to their control group.

Although results seem to suggest an association between serious vitamin D deficiency and an increased risk of dementia, no definitive conclusions can be drawn regarding whether or not lack of sunlight exposure increases a person’s risk of developing dementia. Vitamin D only acts as surrogate parameter for sunlight exposure and our comprehensive searches did not yield any studies on sunlight exposure and dementia risk. Therefore, we had to draw on studies examining the relationship between vitamin D and dementia risk.

Generally, cohort studies can provide data for more definitive conclusions than case-control studies but due to the observational nature of the study designs, no definite conclusion on causality can be drawn in this case. The relationship between lack of vitamin D and the risk of dementia can still be attributed to unknown or residual confounding even though potential confounders have been comprehensively controlled for in most of the analyses. The studies included in this systematic review adjusted extensively for potential confounders. Most of them accounted for physiological (age, gender, BMI), socioeconomic (e.g. education level), and lifestyle factors (smoking, physical activity) as well as comorbidities (diabetes, hypertension). Presence of the ApoE ε4 genotype was only considered by Graf et al. [27].

Another methodological shortcoming compromising the validity of the data is the use of single serum 25(OH)D measurements taken at baseline to represent long-term exposure in all studies [25–30]. As has been demonstrated by several studies, serum 25(OH)D concentrations vary over time within individuals [35, 36], and levels fluctuate seasonally throughout the year due to variances in sunlight exposure [37, 38]. The latter was accounted for in analyses by four studies [26, 28–30] (see Additional file 2: Figure S2).

Other causes of heterogeneity that have been highlighted in previous systematic reviews [11, 12] include age, genetic factors, and method for determining serum vitamin D concentrations. Among the included studies, Graf et al.’s [27] was the only one that utilised a cohort of elderly hospitalised patients, partly suffering from mild cognitive impairment at baseline, and which assessed presence of the ApoE ε4 genotype. However, the effect of its removal on the results of the meta-analysis was negligibly small (see Additional file 2: Figure S3).

Likewise, intra- and inter-rater reliability is reported to differ between methods for determining serum vitamin D concentrations [11, 12]. There is still an ongoing debate regarding the method of choice but a recent comparison between liquid chromatography– tandem mass spectrometry (LC-MS/MS) methods and immunoassays showed variable performance of immunoassays apart from the radioimmunoassay that achieved a performance similar to LC-MS/MS [39]. This could explain why sensitivity analyses contrasting immunoassays and LC-MS/MS methods did not markedly alter the results (see Additional file 2: Figure S4). The systematic review by Balion et al. [11] found significantly greater difference in vitamin D concentrations between Alzheimer’s disease and control groups in studies using competitive protein binding assay (CBPA) than in studies using radioimmunoassay (RIA) or enzyme-linked immunosorbent assay (ELISA), thereby demonstrating variability in immunoassay methods.

We further expected to see differences in dementia incidence and hospitalisation due to dementia as endpoints. Both studies [28, 30] using dementia hospitalisation as endpoints emphasised that cases with dementia events leading to hospitalisation were most likely to be more severe and less frequent than those with dementia incidence identified by experts or registries. However, our sensitivity analysis did not show great differences when removing the studies that used dementia hospitalisation as endpoint (see Additional file 2: Figure S5).

### Strength and limitations of the review

The strength of our review is that we objectively and systematically investigated the association between vitamin D as surrogate for sunlight exposure and dementia risk. We searched multiple scientific databases, hand-searched reference lists and contacted authors to receive data which classified vitamin D according to the cut-off values we required for pooling the data. Notwithstanding, some potential limitations of the review process exist. Despite intensive searches, relevant publications may have been missed. Exclusion of publications written in languages other than English or German could have introduced bias. Although some studies included multi-ethnic populations [29] or explicitly compared white to black populations [30], the majority of participants in the included studies were white, thereby limiting the applicability of our findings to other ethnic groups. Finally, the strength of our conclusion is limited by the very low quality of evidence available for our research question of interest. The identified articles did not include a study that assessed the direct relationship between sunlight exposure and dementia risk.

## Conclusions

The findings of this systematic review are consistent with the hypothesis that low vitamin D levels might contribute to the development of dementia. However, the strength of this conclusion is very low due to several methodological issues such as the possibility of residual confounding, the lack of repeated vitamin D measurements, and the indirectness of the association between sunlight exposure and dementia risk by using vitamin D as surrogate. Further studies examining the indirect and direct relationship between sunlight exposure and dementia risk are needed. Such research should involve large-scale cohort studies with homogeneous groups and repeated assessments of vitamin D concentrations or sunlight exposure in relation to dementia.

## Additional files

Additional file 1: Search strategy of full research report. (DOCX 46 kb)
Additional file 2: Sensitivity analysis. **Figure S1.** Knekt et al. [1] removed due to use of different cut-offs to classify vitamin D; **Figure S2.** Graf et al. [2] removed for reasons of no adjustment for seasonal vitamin D changes, adjustment for presence of ApoE ε4 genotype, elderly population, and a population with partly mild cognitive impairments at baseline; **Figure S3.** Graf et al. [2] and Littlejohns et al. [3] removed due to older populations; **Figure S4.** Littlejohns et al. [3] and Schneider et al. [4] removed due to use of liquid chromatography-tandem mass spectrometry (LC-MS/MS) to measure serum vitamin D concentrations; **Figure S5.** Knekt et al. [1] and Schneider et al. [4] removed due to use of dementia leading to hospitalisation as endpoint. (DOCX 152 kb)

## Abbreviations

BMI: Body mass index
CBPA: Competitive protein binding assay
CI: Confidence interval
ELISA: Enzyme linked immunosorbent assay
LC-MS/MS: Liquid chromatography– tandem mass spectrometry
MMSE: Mini-Mental State Examination
NOS: Newcastle-Ottawa Scale
OR: Odds ratio
PRISMA: Preferred Reporting Items for Systematic Reviews and Meta-Analyses
PROSPERO: International Prospective Register of Systematic Reviews
RIA: Radioimmunoassay
SD: Standard deviation
VDR: 1,25-dihydroxyvitamin D3 receptor
