# Supplementary material for: Vitamin D deficiency as a risk factor for dementia: a systematic review and meta-analysis
Source: BMC Geriatr. 2017 Jan 13;17:16. doi: 10.1186/s12877-016-0405-0 (PMC5237198; doi:10.1186/s12877-016-0405-0)
Supplement: Additional file 1: — Search strategy of full research report. (DOCX 46 kb) [file 12877_2016_405_MOESM1_ESM.docx]

# Additional file 1: Search strategy of full research report

# Pubmed

Research question 1 09.10.2014

| Search | Query | Items found |
| --- | --- | --- |
| #1 | Search "Dementia"[Mesh:NoExp] OR "Alzheimer Disease"[Mesh] OR "Dementia, Vascular"[Mesh] OR "Frontotemporal Lobar Degeneration"[Mesh] OR "Kluver-Bucy Syndrome"[Mesh] OR "Lewy Body Disease"[Mesh] OR "Mild Cognitive Impairment"[Mesh] OR mild cognitive impairment[tiab] OR dementia[tiab] OR alzheimer[tiab] | 132392 |
| #2 | Search "Sunlight"[Mesh] OR "Photoperiod"[Mesh] | 87808 |
| #3 | Search sunlight[tiab] OR daylight[tiab] OR natural light*[tiab] OR daytime light*[tiab] | 12225 |
| #4 | Search "Vitamin D"[Mesh] OR "vitamin d"[tiab] OR Vitamin D2[tiab] OR Vitamin D3[tiab] OR Cholecalciferol*[tiab] OR colecalciferol*[tiab] OR Dihydroxycholecalciferol*[tiab] OR 25 hydroxyvitamin D[tiab] OR 25 OHD[tiab] OR 25 OH vitamin D[tiab] OR 25 hydroxy vit. D[tiab] OR 25 Hydroxycalciferol[tiab] OR 25 hydroxycholecalciferol[tiab] | 61351 |
| #5 | Search "Vitamin D Deficiency"[Mesh] OR Vitamin D deficien*[tiab] | 22791 |
| #6 | Search light exposure[tiab] | 4383 |
| #7 | Search #2 OR #3 OR #4 OR #5 OR #6 | 165096 |
| #8 | Search (#1 AND #7) | 376 |
| #9 | Search "Animals"[Mesh] NOT "Humans"[Mesh] | 3935996 |
| #10 | Search (#8 NOT #9) | 361 |
| #11 | Search ("Infant"[Mesh] OR "Child"[Mesh] OR "Adolescent"[Mesh]) NOT "Adult"[Mesh] | 1513110 |
| #12 | Search (#10 NOT #11) | 359 |
| #13 | Search #12 NOT ("Editorial" [Publication Type] OR "Comment" [Publication Type] OR "Ephemera" [Publication Type]) | 339 |
| #14 | Search #13 AND 1990:2014[dp] | 307 |

Key questions 2 and 3 17.06.2014

| Search | Query | Items found |
| --- | --- | --- |
| #1 | Search ("Nursing Homes"[Mesh] OR "Residential Facilities"[Mesh:NoExp] OR nursing home*[tiab] OR long-term care facilit*[tiab]) | 45319 |
| #2 | Search ("Aged"[Mesh] OR elderly[tiab] OR geriatric[tiab] OR older[tiab] OR aged[tiab] OR elder[tiab]) | 2688364 |
| #3 | Search (#1 AND #2) | 26623 |
| #4 | Search ("Homes for the Aged"[Mesh] OR "Housing for the Elderly"[Mesh] OR geriatric facilit*[tiab] OR retirement home*[tiab]) | 12447 |
| #5 | Search (#3 OR #4) | 32110 |
| #6 | Search ("Sunlight"[Mesh] OR "Photoperiod"[Mesh]) | 86773 |
| #7 | Search (sunlight[tiab] OR daylight[tiab] OR natural light*[tiab] OR daytime light*[tiab]) | 11922 |
| #8 | Search (("Vitamin D"[Mesh] OR "vitamin d"[tiab]) AND ("Light"[Mesh] OR light[tiab])) | 2885 |
| #9 | Search ("Lighting"[Mesh] OR "Light"[Mesh:NoExp]) | 88436 |
| #10 | Search (artificial light*[tiab] OR ambient light*[tiab] OR ambient bright light*[tiab] OR high-intensity light*[tiab]) | 2335 |
| #11 | Search ("Phototherapy"[Mesh] OR phototherap*[tiab] OR light therap*[tiab]) | 30308 |
| #12 | Search light exposure[tiab] | 4282 |
| #13 | Search (#6 OR #7 OR #8 OR #9 OR #10 OR #11 OR #12) | 205959 |
| #14 | Search (#5 AND #13) | 143 |
| #15 | Search ("Animals"[Mesh] NOT "Humans"[Mesh]) | 3896935 |
| #16 | Search (#14 NOT #15) | 143 |
| #17 | Search (("Infant"[Mesh] OR "Child"[Mesh] OR "Adolescent"[Mesh]) NOT "Adult"[Mesh]) | 1496589 |
| #18 | Search (#16 NOT #17) | 143 |
| #19 | Search (#18 AND 1990:2014[dp]) | 129 |
| #20 | Search (#19 NOT ("Editorial" [Publication Type] OR "Comment" [Publication Type] OR "Ephemera" [Publication Type])) | 122 |

# Cochrane Library

Key question 1 09.10.2014

| ID | Search | Hits |
| --- | --- | --- |
| #1 | [mh ^Dementia] or [mh "Alzheimer Disease"] or [mh "Dementia, Vascular"] or [mh "Frontotemporal Lobar Degeneration"] or [mh "Kluver-Bucy Syndrome"] or [mh "Lewy Body Disease"] or [mh "Mild Cognitive Impairment"] or ("mild cognitive impairment" or dementia or alzheimer):ti,ab | 8290 |
| #2 | [mh Sunlight] or [mh Photoperiod] or (sunlight or daylight or "light exposure"):ti,ab or ((natural or daytime) next light*):ti,ab | 1319 |
| #3 | [mh "Vitamin D"] or ("vitamin d" or "Vitamin D2" or "Vitamin D3" or Cholecalciferol* or colecalciferol* or Dihydroxycholecalciferol*):ti,ab or (25 next ("hydroxyvitamin D" or OHD or "OH vitamin D" or "hydroxy vit. D" or Hydroxycalciferol or hydroxycholecalciferol)):ti,ab | 4010 |
| #4 | [mh "Vitamin D Deficiency"] or ("vitamin D" next deficien*):ti,ab | 680 |
| #5 | #2 or #3 or #4 | 5259 |
| #6 | #1 and #5 Publication Year from 1990 to 2014 | 36 |

Key questions 2 and 3 17.06.2014

| ID | Search | Hits |
| --- | --- | --- |
| #1 | [mh Aged] or (elderly or geriatric or "old people" or aged or elder):ti,ab | 123890 |
| #2 | [mh "Nursing Homes"] or [mh ^"Residential Facilities"] or (nursing next home*):ti,ab or ("long-term care" next facilit*):ti,ab | 2215 |
| #3 | #1 and #2 | 1096 |
| #4 | [mh "Homes for the Aged"] or [mh "Housing for the Elderly"] or (geriatric next facilit*):ti,ab or (retirement next home*):ti,ab or ((home* or housing) near/3 (aged or elderly)):ti,ab | 879 |
| #5 | #3 or #4 | 1585 |
| #6 | [mh Sunlight] or [mh Photoperiod] or [mh lighting] or (sunlight or daylight or "light exposure" or lighting):ti,ab or ((natural or daytime) next light*):ti,ab | 9113 |
| #7 | ([mh "Vitamin D"] or "vitamin d":ti,ab) and ([mh light] or light:ti,ab) | 111 |
| #8 | [mh ^light] or ((artificial or ambient or high-intensity) near/2 light*):ti,ab | 586 |
| #9 | [mh phototherapy] or phototherap*:ti,ab or (light next therap*):ti,ab | 2831 |
| #10 | #6 or #7 or #8 or #9 | 10999 |
| #11 | #5 and #10 Publication Year from 1990 to 2014 | 33 |

# Embase

Key question 1 09.10.2014

| No. | Query | Results |
| --- | --- | --- |
| #1 | 'dementia'/de OR 'alzheimer disease'/exp OR 'diffuse lewy body disease'/exp OR 'frontotemporal dementia'/exp OR 'kluver bucy syndrome'/exp OR 'mixed depression and dementia'/exp OR 'multiinfarct dementia'/exp OR 'senile dementia'/exp OR 'mild cognitive impairment'/exp | 195588 |
| #2 | dementia:ab,ti OR 'mild cognitive impairment':ab,ti OR alzheimer:ab,ti | 179050 |
| #3 | #1 OR #2 | 223821 |
| #4 | 'sunlight'/exp OR 'photoperiodicity'/de OR 'brightness'/exp OR 'light'/de | 81266 |
| #5 | sunlight:ab,ti OR daylight:ab,ti OR ((natural OR daytime) NEXT/1 light*):ab,ti | 14145 |
| #6 | 'vitamin d'/de OR '25 hydroxyvitamin d'/exp OR 'colecalciferol'/exp OR 'dihydroxycolecalciferol'/exp OR 'vitamin d derivative'/exp OR 'vitamin d':ab,ti OR 'vitamin d2':ab,ti OR 'vitamin d3':ab,ti OR cholecalciferol*:ab,ti OR colecalciferol*:ab,ti OR dihydroxycholecalciferol*:ab,ti OR (25 NEXT/0 ('hydroxyvitamin d' OR ohd OR 'oh vitamin d' OR 'hydroxy vit. d' OR hydroxycalciferol OR hydroxycholecalciferol)):ab,ti | 87666 |
| #7 | 'vitamin d deficiency'/exp OR ('vitamin d' NEXT/0 deficien*):ab,ti | 16080 |
| #8 | 'light exposure'/exp OR 'light exposure':ab,ti | 9212 |
| #9 | #4 OR #5 OR #6 OR #7 OR #8 | 182739 |
| #10 | #3 AND #9 | 1190 |
| #11 | #10 NOT ('animal'/exp NOT 'human'/exp) | 1139 |
| #12 | #11 NOT ('juvenile'/exp NOT 'adult'/exp) | 1130 |
| #13 | #12 AND [1990-2014]/py NOT ('editorial'/it OR 'letter'/it OR 'note'/it) | 983 |

Key questions 2 and 3 17.06.2014

| No. | Query | Results |
| --- | --- | --- |
| #1 | 'nursing home'/exp OR 'residential home'/exp OR 'home care'/de OR 'residential care'/exp OR 'institutional care'/de OR 'home for the aged'/exp OR 'elderly care'/exp | 154079 |
| #2 | (('long term care' OR geriatric OR retirement OR nursing) NEXT/1 (home* OR facilit* OR housing)):ab,ti | 33684 |
| #3 | #1 OR #2 | 162855 |
| #4 | 'geriatric patient'/exp | 15044 |
| #5 | ((elderly OR geriatric OR aged OR elder OR older) NEXT/1 (person* OR people OR patient* OR resident*)):ab,ti | 156295 |
| #6 | 'patient'/de OR 'nursing home patient'/exp AND (elderly:ab,ti OR aged:ab,ti OR elder:ab,ti OR older:ab,ti) | 87602 |
| #7 | 'aged'/exp | 2123221 |
| #8 | #4 OR #5 OR #6 OR #7 | 2231872 |
| #9 | #3 AND #8 | 80285 |
| #10 | 'sunlight'/exp OR 'photoperiodicity'/de OR 'brightness'/exp OR 'light'/de OR OR 'phototherapy'/de OR 'light therapy':ab,ti | 100834 |
| #11 | sunlight:ab,ti OR daylight:ab,ti OR ((natural OR daytime) NEXT/1 light*):ab,ti | 13824 |
| #12 | 'vitamin d'/exp OR 'vitamin d':ab,ti AND (light*:ab,ti OR 'light'/exp) | 3328 |
| #13 | 'illumination'/exp OR 'light exposure'/exp OR 'light exposure':ab,ti | 25234 |
| #14 | ((artificial OR ambient OR 'high intensity' OR bright) NEXT/2 light*):ab,ti | 5332 |
| #15 | #10 OR #11 OR #12 OR #13 OR #14 | 126965 |
| #16 | #9 AND #15 | 234 |
| #17 | #16 NOT ('animal'/exp NOT 'human'/exp) | 234 |
| #18 | #17 NOT ('juvenile'/exp NOT 'adult'/exp) | 234 |
| #19 | #18 AND [1990-2014]/py | 220 |
| #20 | #19 NOT ('editorial'/it OR 'letter'/it OR 'note'/it) | 204 |

# Scopus

Key question 1 09.10.2014

| Query | Results |
| --- | --- |
| ((TITLE-ABS-KEY(sunlight OR daylight) OR TITLE-ABS-KEY((natural OR daytime) PRE/1 light*) OR TITLE-ABS-KEY("light exposure") OR TITLE-ABS-KEY("vitamin d" OR "Vitamin D2" OR "Vitamin D3" OR Cholecalciferol* OR colecalciferol* OR Dihydroxycholecalciferol*) OR TITLE-ABS-KEY(25 PRE/0 ("hydroxyvitamin D" OR OHD OR "OH vitamin D" OR "hydroxy vit. D" OR Hydroxycalciferol OR hydroxycholecalciferol)) OR TITLE-ABS-KEY("vitamin d" PRE/0 deficien*)) AND PUBYEAR > 1989) AND (TITLE-ABS-KEY(dementia OR Alzheimer OR "mild cognitive impairment") AND PUBYEAR > 1989) AND NOT (KEY(Animal OR Animals OR nonhuman) AND NOT KEY(Human OR Humans)) AND ( EXCLUDE(DOCTYPE,"ed" ) OR EXCLUDE(DOCTYPE,"le" ) OR EXCLUDE(DOCTYPE,"no" ) ) | 872 |

Key questions 2 and 3 17.06.2014

| Search | Results |
| --- | --- |
| ((TITLE-ABS-KEY((nursing OR residential OR "old people" OR "long term care" OR geriatric OR retirement) PRE/1 (home* OR housing OR facilit*))) OR (TITLE-ABS-KEY("home for the aged" OR "elderly care" OR "home for the elderly"))) AND (TITLE-ABS-KEY((elderly OR geriatric OR aged OR elder OR older) PRE/1 (person* OR people OR patient* OR resident*))) AND ((TITLE-ABS-KEY(sunlight OR daylight) OR TITLE-ABS-KEY((natural OR daytime) PRE/1 light*) OR TITLE-ABS-KEY("light exposure") OR TITLE-ABS-KEY("vitamin d" AND light) OR TITLE-ABS-KEY((artificial OR ambient OR "high intensity" OR bright) PRE/2 light*) OR TITLE-ABS-KEY(phototherapy OR "light therapy"))) AND (EXCLUDE(PUBYEAR, 1988) OR EXCLUDE(PUBYEAR, 1987) OR EXCLUDE(PUBYEAR, 1985) OR EXCLUDE(PUBYEAR, 1984) OR EXCLUDE(PUBYEAR, 1981) OR EXCLUDE(PUBYEAR, 1979) OR EXCLUDE(PUBYEAR, 1978) OR EXCLUDE(PUBYEAR, 1988) OR EXCLUDE(PUBYEAR, 1987) OR EXCLUDE(PUBYEAR, 1985) OR EXCLUDE(PUBYEAR, 1984) OR EXCLUDE(PUBYEAR, 1981) OR EXCLUDE(PUBYEAR, 1979) OR EXCLUDE(PUBYEAR, 1978)) AND (EXCLUDE(DOCTYPE, "le") OR EXCLUDE(DOCTYPE, "ed") OR EXCLUDE(DOCTYPE, "no")) | 69 |

# Web of Science

Key question 09.10.2014

| Set | Results | Search |
| --- | --- | --- |
|  | Approximately | Search language=Auto |
|  | 595,636 | Timespan=1990-2014 |
| # 1 |  | TOPIC: (dementia OR alzheimer OR "mild cognitive impairment") |
|  | Approximately | Search language=Auto |
|  | 141,281 | Timespan=1990-2014 |
| # 2 |  | TOPIC: (sunlight OR daylight) OR TOPIC: ((natural OR daytime) NEAR/1 light*) OR TOPIC: ("light exposure") |
|  | Approximately | Search language=Auto |
|  | 168,798 | Timespan=1990-2014 |
| # 3 |  | TOPIC: ("vitamin d" OR "Vitamin D2" OR "Vitamin D3" OR Cholecalciferol* OR colecalciferol* OR Dihydroxycholecalciferol*) OR TOPIC: ("25 hydroxyvitamin D" OR "25 OHD" OR "25 OH vitamin D" OR "25 hydroxy vit. D" OR "25 Hydroxycalciferol" OR "25 hydroxycholecalciferol") OR TOPIC: ("vitamin d" NEAR/0 deficien*) |
|  | Approximately | Search language=Auto |
|  | 304,548 | Timespan=1990-2014 |
| # 4 |  | #3 OR #2 |
|  |  | Search language=Auto |
|  |  | Timespan=1990-2014 |
| # 5 | 757 | #4 AND #1 |
|  |  | Search language=Auto |
|  |  | Timespan=1990-2014 |
|  |  | Refined by: [excluding] Databases: ( MEDLINE OR DIIDW OR ZOOREC ) |
| # 6 | 127 | #4 AND #1 |
|  |  | Search language=Auto |
|  |  | Timespan=1990-2014 |
|  |  | Refined by: [excluding] Databases: ( MEDLINE OR DIIDW OR ZOOREC ) AND [excluding] DOCUMENT TYPES: ( EDITORIAL OR LETTER ) |
| # 7 | 115 | #4 AND #1 |

Key questions 2 and 3 17.06.2014

| Set | Results | Search |
| --- | --- | --- |
|  |  | Search language=English |
|  |  | Timespan=1990-2014 |
| # 2 | 1,793 | TOPIC: ("home for the aged" OR "elderly care" OR "home for the elderly") |
|  |  |  |
|  |  | Search language=English |
|  | 119,026 | Timespan=1990-2014 |
| # 3 | Approximately | #2 OR #1 |
|  |  |  |
|  |  | Search language=English |
|  | 1,056,475 | Timespan=1990-2014 |
| # 4 | Approximately | TOPIC: ((elderly OR geriatric OR aged OR elder OR older) NEAR/1 (person* OR people OR patient* OR resident*)) |
|  |  |  |
|  |  | Search language=English |
|  | 28,553 | Timespan=1990-2014 |
| # 5 | Approximately | #4 AND #3 |
|  |  |  |
|  |  | Search language=English |
|  | 201,297 | Timespan=1990-2014 |
| # 6 | Approximately | TOPIC: (sunlight OR daylight) *OR* TOPIC: ((natural OR daytime) NEAR/1 light*) *OR* TOPIC: ("light exposure") *OR* GROUP AUTHOR: ("vitamin D" AND light) *OR* TOPIC: (phototherapy OR "light therapy") *OR* TOPIC: ((artificial OR ambient OR "high intensity" OR bright) NEAR/2 light*) |
|  |  |  |
|  |  | Search language=English |
|  |  | Timespan=1990-2014 |
| # 7 | 96 | #6 AND #5 |
|  |  |  |
|  |  | Search language=Auto |
|  |  | Timespan=1990-2014 |
|  |  | Refined by: [excluding] Databases: ( MEDLINE ) |
| # 8 | 16 | #6 AND #5 |
|  |  |  |
| # 1 | Approximately | TOPIC: ((nursing OR residential OR "old people" OR "long term care" OR geriatric OR retirement) NEAR/1 (home* OR housing OR facilit*)) |
|  | 116,752 | Timespan=1990-2014 |
|  |  | Search language=English |

# ICONDA

Key question 1 17.06.2014

| # | Suchen | Ergebnisse |
| --- | --- | --- |
| 1 | dementia.de. or dementia.mp. | 92 |
| 2 | demenz.mp. | 17 |
| 3 | Alzheimer's disease.de. or alzheimer.mp. | 25 |
| 4 | 1 or 2 or 3 | 120 |
| 5 | lighting.de. or sunlight.mp. | 7543 |
| 6 | daylight.mp. or daylight.de. or daylighting.de. | 5098 |
| 7 | ((natural or daytime) adj light*).mp. [mp=abstract, heading word, title alternate, title enhancement, title] | 625 |
| 8 | ("light/sun" or daylight usage).de. or tageslicht.mp. | 1463 |
| 9 | sun light.de. or sonnenlicht.mp. | 83 |
| 10 | 5 or 6 or 7 or 8 or 9 | 13059 |
| 11 | 4 and 10 | 11 |

Key questions 2 and 3 17.06.2014

| # | Suchen | Ergebnisse |
| --- | --- | --- |
| 1 | nursing home.mp. or Nursing Homes.de. or nursing home.de. or geriatric home.de. | 1217 |
| 2 | residential home.mp. or residence for the disabled.de. | 282 |
| 3 | home for the aged.mp. or housing for the elderly.de. | 1483 |
| 4 | (('long term care' or geriatric or retirement or nursing) adj (home* or facilit* or housing)).mp. [mp=abstract, heading word, title alternate, title enhancement, title] | 1405 |
| 5 | (old people's home or home for the elderly or housing for the elderly).de. or altersheim.mp. | 2528 |
| 6 | assisted living.de. or pflegeheim.mp. | 219 |
| 7 | 1 or 2 or 3 or 4 or 5 or 6 | 3607 |
| 8 | lighting.de. or sunlight.mp. | 7543 |
| 9 | daylight.mp. or daylight.de. or daylighting.de. | 5098 |
| 10 | (Lighting or lighting system).de. or natural light.mp. | 8800 |
| 11 | (artificial light or interior lighting or artificial lighting or lighting technology).de. or artificial light*.mp. or "light/sun".de. | 3044 |
| 12 | Lighting - Artificial.de. | 62 |
| 13 | ((artificial or ambient or 'high intensity' or bright) adj2 light*).mp. [mp=abstract, heading word, title alternate, title enhancement, title] | 1087 |
| 14 | (light planning or light control or daylight usage).de. or tageslicht.mp. | 1529 |
| 15 | sonnenlicht.mp. | 32 |
| 16 | (illuminating engineering or illuminance).de. or kunstlicht.mp. | 1726 |
| 17 | beleuchtung.mp. | 591 |
| 18 | 8 or 9 or 10 or 11 or 12 or 13 or 14 or 15 or 16 or 17 | 16192 |
| 19 | 7 and 18 | 82 |
| 20 | limit 19 to yr="1990 -Current" | 63 |

# PsycInfo

Key question 1 09.10.2014

| # | Query | Results |
| --- | --- | --- |
| S1 | DE "Dementia" OR DE "Dementia with Lewy Bodies" OR DE "Senile Dementia" OR DE "Vascular Dementia" OR DE "Alzheimer's Disease" | 52,198 |
| S2 | TI ( dementia OR "mild cognitive impairment" OR alzheimer ) OR AB ( dementia OR "mild cognitive impairment" OR alzheimer ) | 68,366 |
| S3 | S1 OR S2 | 70,006 |
| S4 | TX ( sunlight OR daylight ) OR ( (natural OR daytime) W1 light* ) | 1,512 |
| S5 | TX "light exposure" | 677 |
| S6 | ( TX ("vitamin d" OR "Vitamin D2" OR "Vitamin D3" OR Cholecalciferol* OR colecalciferol* OR Dihydroxycholecalciferol*) ) OR ( TX (25 W0 ("hydroxyvitamin D" OR OHD OR "OH vitamin D" OR "hydroxy vit. D" OR Hydroxycalciferol OR hydroxycholecalciferol)) ) OR TX ("vitamin d" PRE/0 deficien*) | 1,119 |
| S7 | S4 OR S5 OR S6 | 3,164 |
| S8 | (S4 OR S5 OR S6) AND (S3 AND S7) Limiters - Publication Year: 1990-2014 | 128 |

Key questions 2 and 3 18.06.2014

| # | Query | Results |
| --- | --- | --- |
| S1 | DE "Nursing Homes" OR DE "Assisted Living" OR DE "Group Homes" OR DE "Retirement Communities" OR DE "Residential Care Institutions" OR DE "Treatment Facilities" | 17,275  Formularende |
| S2 | TX ("long term care" OR geriatric OR retirement OR nursing) W1 (home* OR facilit* OR housing) | 15,599 |
| S3 | S1 OR S2 | 22,331 |
| S4 | DE "Geriatric Patients" | 10,829 |
| S5 | DE "Patients" AND (TX (elderly OR aged OR older OR elder)) | 11,802 |
| S6 | TI ( (elderly OR geriatric OR aged OR elder OR older) W1 (person* OR people OR patient* OR resident*) ) OR AB ( (elderly OR geriatric OR aged OR elder OR older) W1 (person* OR people OR patient* OR resident*) ) | 33,152 |
| S7 | S4 OR S5 OR S6 | 49,657 |
| S8 | S3 AND S7 | 4,467 |
| S9 | TX ( sunlight OR daylight ) OR ( (natural OR daytime) W1 light* ) | 1,484 |
| S10 | TX "vitamin d" AND light | 52 |
| S11 | TX "light exposure" | 659 |
| S12 | DE "Illumination" OR DE "Phototherapy" | 8,194 |
| S13 | TX (artificial OR ambient OR "high intensity" OR bright) W2 light* | 1,767 |
| S14 | TX "light therapy" | 678 |
| S15 | S9 OR S10 OR S11 OR S12 OR S13 OR S14 | 10,532 |
| S16 | S8 AND S15 | 12 |

Update Search Key question 1 15.10.2015

Pubmed

| Search | Query | Items found |
| --- | --- | --- |
| #1 | Search "Dementia"[Mesh:NoExp] OR "Alzheimer Disease"[Mesh] OR "Dementia, Vascular"[Mesh] OR "Frontotemporal Lobar Degeneration"[Mesh] OR "Kluver-Bucy Syndrome"[Mesh] OR "Lewy Body Disease"[Mesh] OR "Mild Cognitive Impairment"[Mesh] | 109216 |
| #2 | Search mild cognitive impairment[tiab] OR dementia[tiab] OR alzheimer[tiab] | 91803 |
| #3 | Search (#1 OR #2) | 142999 |
| #4 | Search "Sunlight"[Mesh] OR "Photoperiod"[Mesh] | 91140 |
| #5 | Search sunlight[tiab] OR daylight[tiab] OR natural light*[tiab] OR daytime light*[tiab] | 13738 |
| #6 | Search "Vitamin D"[Mesh] | 45794 |
| #7 | Search vitamin d[tiab] OR Vitamin D2[tiab] OR Vitamin D3[tiab] OR Cholecalciferol*[tiab] OR colecalciferol*[tiab] OR Dihydroxycholecalciferol*[tiab] OR 25 hydroxyvitamin D[tiab] OR 25 OHD[tiab] OR 25 OH vitamin D[tiab] OR 25 hydroxy vit. D[tiab] OR 25 Hydroxycalciferol[tiab] OR 25 hydroxycholecalciferol[tiab] | 51927 |
| #8 | Search "Vitamin D Deficiency"[Mesh] | 21257 |
| #9 | Search light exposure[tiab] | 4732 |
| #10 | Search (#4 OR #5 OR #6 OR #7 OR #8 OR #9) | 174030 |
| #11 | Search (#3 AND #10) | 434 |
| #12 | Search "Animals"[Mesh] NOT "Humans"[Mesh] | 4050309 |
| #13 | Search (#11 NOT #12) | 416 |
| #14 | Search ("Infant"[Mesh] OR "Child"[Mesh] OR "Adolescent"[Mesh]) NOT "Adult"[Mesh] | 1561462 |
| #15 | Search (#13 NOT #14) | 413 |
| #16 | Search #15 NOT ("Editorial" [Publication Type] OR "Comment" [Publication Type] OR "Ephemera" [Publication Type]) | 391 |
| #17 | Search (#16) AND ("2014"[Date - Publication] : "3000"[Date - Publication]) | 89 |

Cochrane Library

| ID | Search | Hits |
| --- | --- | --- |
| #1 | [mh ^Dementia] or [mh "Alzheimer Disease"] or [mh "Dementia, Vascular"] or [mh "Frontotemporal Lobar Degeneration"] or [mh "Kluver-Bucy Syndrome"] or [mh "Lewy Body Disease"] or [mh "Mild Cognitive Impairment"] | 3828 |
| #2 | ("mild cognitive impairment" or dementia or alzheimer):ti,ab,kw | 9700 |
| #3 | #1 or #2 | 9708 |
| #4 | [mh Sunlight] or [mh Photoperiod] | 961 |
| #5 | [mh "Vitamin D"] | 2447 |
| #6 | (sunlight or daylight or "light exposure"):ti,ab or ((natural or daytime) next light*):ti,ab,kw | 542 |
| #7 | ("vitamin d" or "Vitamin D2" or "Vitamin D3" or Cholecalciferol* or colecalciferol* or Dihydroxycholecalciferol*):ti,ab or (25 next ("hydroxyvitamin D" or OHD or "OH vitamin D" or "hydroxy vit. D" or Hydroxycalciferol or hydroxycholecalciferol)):ti,ab,kw | 4512 |
| #8 | [mh "Vitamin D Deficiency"] | 509 |
| #9 | {or #4-#8} | 6503 |
| #10 | #3 and #9 Publication Year from 2014 | 5 |

Embase

| No. | Query | Results |
| --- | --- | --- |
| #1 | 'dementia'/de OR 'alzheimer disease'/exp OR 'diffuse lewy body disease'/exp OR 'frontotemporal dementia'/exp OR 'kluver bucy syndrome'/exp OR 'mixed depression and dementia'/exp OR 'multiinfarct dementia'/exp OR 'senile dementia'/exp OR 'mild cognitive impairment'/exp | 213879 |
| #2 | dementia:ab,ti OR 'mild cognitive impairment':ab,ti OR alzheimer:ab,ti | 197741 |
| #3 | #1 OR #2 | 245637 |
| #4 | 'sunlight'/exp OR 'photoperiodicity'/de OR 'brightness'/exp OR 'light'/de | 87673 |
| #5 | sunlight:ab,ti OR daylight:ab,ti OR ((natural OR daytime) NEXT/1 light*):ab,ti | 15456 |
| #6 | 'vitamin d'/de OR '25 hydroxyvitamin d'/exp OR 'colecalciferol'/exp OR 'dihydroxycolecalciferol'/exp OR 'vitamin d derivative'/exp | 73776 |
| #7 | 'vitamin d':ab,ti OR 'vitamin d2':ab,ti OR 'vitamin d3':ab,ti OR cholecalciferol*:ab,ti OR colecalciferol*:ab,ti OR dihydroxycholecalciferol*:ab,ti OR (25 NEXT/0 ('hydroxyvitamin d' OR ohd OR 'oh vitamin d' OR 'hydroxy vit. d' OR hydroxycalciferol OR hydroxycholecalciferol)):ab,ti | 68957 |
| #8 | 'vitamin d deficiency'/exp | 18718 |
| #9 | 'light exposure'/exp | 7208 |
| #10 | 'light exposure':ab,ti | 5413 |
| #11 | #4 OR #5 OR #6 OR #7 OR #8 OR #9 OR #10 | 199315 |
| #12 | #3 AND #11 | 1352 |
| #13 | #12 NOT ('animal'/exp NOT 'human'/exp) | 1289 |
| #14 | #13 NOT ('groups by age'/exp NOT 'adult'/exp) | 1268 |
| #15 | #14 NOT ('editorial'/it OR 'letter'/it OR 'note'/it) | 1141 |
| #16 | #15 AND [1-9-2014]/sd | 159 |

Scopus

| Query | Results |
| --- | --- |
| ((TITLE-ABS-KEY(sunlight OR daylight) OR TITLE-ABS-KEY((natural OR daytime) PRE/1 light*) OR TITLE-ABS-KEY("light exposure") OR TITLE-ABS-KEY("vitamin d" OR "Vitamin D2" OR "Vitamin D3" OR Cholecalciferol* OR colecalciferol* OR Dihydroxycholecalciferol*) OR TITLE-ABS-KEY(25 PRE/0 ("hydroxyvitamin D" OR OHD OR "OH vitamin D" OR "hydroxy vit. D" OR Hydroxycalciferol OR hydroxycholecalciferol))) AND PUBYEAR > 2014) AND (TITLE-ABS-KEY(dementia OR Alzheimer OR "mild cognitive impairment") AND PUBYEAR > 2014) AND NOT (KEY(Animal OR Animals OR nonhuman) AND NOT KEY(Human OR Humans)) AND ( EXCLUDE(DOCTYPE,"ed" ) OR EXCLUDE(DOCTYPE,"le" ) OR EXCLUDE(DOCTYPE,"no" ) ) | 72 |

Web of Science

| Set | Results | Search |
| --- | --- | --- |
|  |  | Search language=Auto |
|  | 89,653 | Timespan=2014-2015 |
| # 1 | Approximately | TOPIC: (dementia OR alzheimer OR "mild cognitive impairment") |
|  |  | Search language=Auto |
|  | 24,293 | Timespan=2014-2015 |
| # 2 | Approximately | TOPIC: (sunlight OR daylight OR "light exposure") OR TOPIC: ((natural OR daytime) NEAR/1 light*) |
|  |  | Search language=Auto |
|  | 27,262 | Timespan=2014-2015 |
| # 3 | Approximately | TOPIC: ("vitamin d" OR "Vitamin D2" OR "Vitamin D3" OR Cholecalciferol* OR colecalciferol* OR Dihydroxycholecalciferol*) OR TOPIC: ("25 hydroxyvitamin D" OR "25 OHD" OR "25 OH vitamin D" OR "25 hydroxy vit. D" OR "25 Hydroxycalciferol" OR "25 hydroxycholecalciferol") |
|  |  | Search language=Auto |
|  | 50,805 | Timespan=2014-2015 |
| # 4 | Approximately | #3 OR #2 |
|  |  | Search language=Auto |
|  |  | Timespan=2014-2015 |
| # 5 | 229 | #4 AND #1 |
|  |  | Search language=Auto |
|  |  | Timespan=2014-2015 |
|  |  | Refined by: [excluding] Databases: ( DIIDW OR MEDLINE ) |
| # 6 | 23 | #4 AND #1 |
|  |  | Search language=Auto |
|  |  | Timespan=2014-2015 |
|  |  | Refined by: [excluding] Databases: ( DIIDW OR MEDLINE ) AND [excluding] DOCUMENT TYPES: ( NEWS OR EDITORIAL ) |
| # 7 | 21 | #4 AND #1 |

PsycINFO

| # | Query | Results |
| --- | --- | --- |
| S1 | DE "Dementia" OR DE "Dementia with Lewy Bodies" OR DE "Senile Dementia" OR DE "Vascular Dementia" OR DE "Alzheimer's Disease" |  |
| S2 | TI ( dementia OR "mild cognitive impairment" OR alzheimer ) OR AB ( dementia OR "mild cognitive impairment" OR alzheimer ) | 74,785 |
| S3 | S1 OR S2 | 76,651 |
| S4 | TX ( sunlight OR daylight ) OR ( (natural OR daytime) W1 light* ) | 1,626 |
| S5 | TX "light exposure" | 750 |
| S6 | TX ("vitamin d" OR "Vitamin D2" OR "Vitamin D3" OR Cholecalciferol* OR colecalciferol* OR Dihydroxycholecalciferol*) ) OR ( TX (25 W0 ("hydroxyvitamin D" OR OHD OR "OH vitamin D" OR "hydroxy vit. D" OR Hydroxycalciferol OR hydroxycholecalciferol)) ) | 1,35 |
| S7 | S4 OR S5 OR S6 | 3,563 |
| S8 | S3 AND S7 | 158 |
| S9 | S3 AND S7 Limiters - Publication Year: 2014- | 43 |
