# Supplementary material for: Vitamin D deficiency as a risk factor for dementia: a systematic review and meta-analysis
Source: BMC Geriatr. 2017 Jan 13;17:16. doi: 10.1186/s12877-016-0405-0 (PMC5237198; doi:10.1186/s12877-016-0405-0)
Supplement: Additional file 2: — Sensitivity analysis. Figure S1. Knekt et al. [1] removed due to use of different cut-offs to classify vitamin D; Figure S2. Graf et al. [2] removed for reasons of no adjustment for seasonal vitamin D changes, adjustment for presence of ApoE ε4 genotype, elderly population, and a population with partly mild cognitive impairments at baseline; Figure S3. Graf et al. [2] and Littlejohns et al. [3] removed due to older populations; Figure S4. Littlejohns et al. [3] and Schneider et al. [4] removed due to use of liquid chromatography-tandem mass spectrometry (LC-MS/MS) to measure serum vitamin D concentrations; Figure S5. Knekt et al. [1] and Schneider et al. [4] removed due to use of dementia leading to hospitalisation as endpoint. (DOCX 152 kb) [file 12877_2016_405_MOESM2_ESM.docx]

**Additional File 1: Sensitivity analyses**

Figure 1: Knekt et al. [1] removed due to use of different cut-offs to classify vitamin D

100%

Total

**Study name**

**Point estimate and 95% CI**

Point

Lower

Upper

Relative

estimate

limit

limit

Weight (%)

Afzal 2014

1.27

1.01

1.60

78.52

Graf 2014

1.35

0.39

4.64

2.85

Littlejohns 2014

2.25

1.23

4.12

11.82

Schneider 2014

1.44

0.65

3.20

6.82

1.37

1.11

1.69

**0.1**

**0.2**

**0.5**

**1**

**2**

**5**

**10**

**No Vit. D deficiency**

**Serious Vit. D deficiency**

**Random effects meta-analysis; I² = 1%**

**Statistics for each study**

Figure 2: Graf et al. [2] removed for reasons of no adjustment for seasonal vitamin D changes, adjustment for presence of ApoE ε4 genotype, elderly population, and a population with partly mild cognitive impairments at baseline

100%

Total

**Study name**

**Point estimate and 95% CI**

Point

Lower

Upper

Relative

estimate

limit

limit

Weight (%)

Afzal 2014

1.27

1.01

1.60

46.87

Knekt 2014

2.08

1.20

3.60

21.60

Littlejohns 2014

2.25

1.23

4.12

18.99

Schneider 2014

1.44

0.65

3.20

12.54

1.60

1.17

2.19

**0.1**

**0.2**

**0.5**

**1**

**2**

**5**

**10**

**No Vit. D deficiency**

**Serious Vit. D deficiency**

**Random effects meta-analysis; I² = 40%**

**Statistics for each study**

Figure 3: Graf et al. [2] and Littlejohns et al. [3] removed due to older populations

Total

100%

**Study name**

**Point estimate and 95% CI**

Point

Lower

Upper

Relative

estimate

limit

limit

Weight (%)

Afzal 2014

1.27

1.01

1.60

65.26

Knekt 2014

2.08

1.20

3.60

22.66

Schneider 2014

1.44

0.65

3.20

12.08

1.44

1.07

1.93

**0.1**

**0.2**

**0.5**

**1**

**2**

**5**

**10**

**No Vit. D deficiency**

**Serious Vit. D deficiency**

**Random effects meta-analysis; I² = 25%**

**Statistics for each study**

Figure 4: Littlejohns et al. [3] and Schneider et al. [4] removed due to use of liquid chromatography-tandem mass spectrometry (LC-MS/MS) to measure serum vitamin D concentrations

100%

Total

**Study name**

**Point estimate and 95% CI**

Point

Lower

Upper

Relative

estimate

limit

limit

Weight (%)

Afzal 2014

1.27

1.01

1.60

68.37

Graf 2014

1.35

0.39

4.64

6.22

Knekt 2014

2.08

1.20

3.60

25.42

1.45

1.05

1.98

**0.1**

**0.2**

**0.5**

**1**

**2**

**5**

**10**

**No Vit. D deficiency**

**Serious Vit. D deficiency**

**Random effects meta-analysis; I² = 24%**

**Statistics for each study**

Figure 5: Knekt et al. [1] and Schneider et al. [4] removed due to use of dementia leading to hospitalisation as endpoint

100%

Total

**Study name**

**Point estimate and 95% CI**

Point

Lower

Upper

Relative

estimate

limit

limit

Weight (%)

Afzal 2014

1.27

1.01

1.60

64.61

Graf 2014

1.35

0.39

4.64

8.49

Littlejohns 2014

2.25

1.23

4.12

26.89

1.49

1.02

2.18

**0.1**

**0.2**

**0.5**

**1**

**2**

**5**

**10**

**No Vit. D deficiency**

**Serious Vit. D deficiency**

**Random effects meta-analysis; I² = 33%**

**Statistics for each study**
